# Successful Transition in Rare Metabolic Bone Diseases: One-Year Outcomes of a Multidisciplinary Pediatric–Adult Program

**DOI:** 10.3390/medicina62071336

**Published:** 2026-07-11

**Authors:** Müge Yaşar, Özen Öz Gül, Hatice Nursoy, Filiz Mercan Sarıdaş, Erhan Hocaoğlu, Kadircan Karatoprak, Yasemin Denkboy Öngen, Erdal Eren, Soner Cander, Canan Ersoy, Erdinç Ertürk

**Affiliations:** 1 Division of Endocrinology and Metabolic Diseases, Department of Internal Medicine, Faculty of Medicine, Bursa Uludağ University, 16059 Bursa, Turkeykaratoprak.kadircan@gmail.com (K.K.);; 2 Division of Pediatric Endocrinology, Department of Pediatrics, Faculty of Medicine, Bursa Uludağ University, 16059 Bursa, Turkey

**Keywords:** transition to adult care, metabolic bone disease, multidisciplinary transition clinic, rare endocrine disorders, continuity of care

## Abstract

*Background and Objectives:* Pediatric-onset metabolic bone diseases, including osteogenesis imperfecta (OI), hypophosphatemic rickets (XLH), hypoparathyroidism, and McCune–Albright syndrome (MAS), require lifelong follow-up because of persistent skeletal fragility, biochemical abnormalities, and functional morbidity extending into adulthood. However, evidence regarding structured transition from pediatric to adult care in these rare disorders remains limited. This study evaluated one-year outcomes of a multidisciplinary transition program for adolescents and young adults with rare metabolic bone diseases. *Materials and Methods:* This retrospective cohort study included 20 patients aged ≥17 years who underwent evaluation through a structured transition pathway consisting of multidisciplinary team meetings, a joint pediatric–adult transition clinic, and subsequent follow-up in adult endocrinology. Demographic, clinical, treatment, and transition-related data were extracted from medical records. The primary outcome was successful transition, defined as at least one adult endocrinology visit within 12 months. Secondary outcomes included attendance at the transition clinic, follow-up continuity, and treatment modifications. *Results:* All patients underwent multidisciplinary evaluation, and 85% attended the joint transition clinic. Successful transfer to adult endocrinology was achieved in 90% (18/20), while regular follow-up during the first year was maintained in 75%. Retention was highest in patients with OI, MAS, XLH, vitamin D-dependent rickets, and DiGeorge syndrome (100%). Greater variability was observed in postoperative and primary hypoparathyroidism. Treatment adjustments were required in 40% of patients, including optimization of phosphate/calcitriol replacement and reassessment of bisphosphonate or burosumab therapy. Three patients were lost to follow-up. No acute transition-related complications were observed. *Conclusions:* In this small exploratory cohort, implementation of a structured multidisciplinary transition pathway was feasible and was accompanied by high transfer and one-year retention rates. Observed differences across diagnostic subgroups should be interpreted cautiously, and larger multicenter comparative studies are needed to evaluate the effectiveness of structured transition frameworks.

## 1. Introduction

Metabolic bone diseases that manifest during childhood—including osteogenesis imperfecta (OI), hypophosphatemic rickets (XLH), hypoparathyroidism, hypophosphatasia (HPP), McCune–Albright syndrome, and vitamin D-dependent rickets—are lifelong conditions with clinical trajectories that extend far beyond adolescence [1,2]. Although initial diagnosis and early management typically occur in pediatric endocrine and metabolic clinics, these disorders frequently persist into adulthood with evolving morbidity. Studies have consistently demonstrated that complications such as recurrent fractures, progressive skeletal deformities, chronic pain, impaired mobility, mineral dysregulation, and reduced quality of life continue or even intensify during adulthood despite earlier pediatric interventions [3,4,5]. For instance, adult patients with OI exhibit a bimodal distribution of fracture risk, with a second peak emerging later in life; similarly, adults with XLH frequently report persistent functional limitations, enthesopathy, and chronic pain despite treatment in childhood [1,3,4,5]. In HPP, misdiagnosis in adulthood may lead to inappropriate antiresorptive therapy, resulting in worsening disease severity [6]. These long-term manifestations highlight the necessity for uninterrupted, developmentally appropriate transition from pediatric to adult care [7,8].

Transition of care is defined as the purposeful, planned movement of adolescents and young adults with chronic conditions from child-centered to adult-oriented healthcare systems. A robust body of literature—from fields such as inflammatory bowel disease, epilepsy, and pediatric rheumatology—demonstrates that transition is not a single event, but a longitudinal process requiring the assessment of readiness, structured education, coordinated communication, and the progressive development of self-management skills [9,10,11]. Yet, despite its recognized importance, transition is often suboptimal across chronic disease groups. Reports from epilepsy and pediatric-onset lupus cohorts reveal strikingly low successful transfer rates (as low as 14–50%) and prolonged gaps in care following pediatric discharge [12,13]. Emotional dependence on pediatric providers, limited experience of adult specialists with rare pediatric-onset conditions, and patients’ inadequate self-management skills have emerged as significant barriers [9,11,12].

In contrast to better-studied chronic diseases, evidence guiding transition in pediatric-onset metabolic bone disorders is extremely limited. These conditions are rare, heterogeneous, and often managed in highly specialized pediatric units, resulting in fragmented adult-care pathways and a lack of standardized transition models [5,7]. Existing knowledge is primarily derived from expert opinion or small narrative reviews, and there is a notable absence of empirical data describing real-world transition practices, attendance rates, continuity of therapy, or disease-specific challenges during the transfer to adult endocrinology care.

Given the lifelong nature of metabolic bone diseases and the increasing recognition that inadequate transition may lead to treatment discontinuity, preventable morbidity, and delayed management of adult complications, there is a critical need to characterize how these patients navigate the transition period. Understanding factors that influence successful transfer, patterns of follow-up in adult clinics, and the feasibility of multidisciplinary transition pathways would provide essential insights for improving long-term outcomes.

The present study aimed to address this gap by evaluating the transition process of adolescents and young adults with metabolic bone diseases within a structured pediatric-to-adult transition program at a tertiary center. The primary objective of this exploratory study was to evaluate the feasibility of a structured multidisciplinary transition pathway and to describe transition outcomes during the first year after transfer to adult care. Our working hypothesis was that implementation of a structured multidisciplinary transition pathway would facilitate successful transfer and continuity of follow-up in adult endocrinology care. By examining real-world participation in multidisciplinary transition meetings, attendance at joint transition clinics, and follow-up patterns in adult endocrinology, this study sought to identify the strengths, challenges, and unmet needs within the current model rather than to establish the effectiveness of the transition program. The present study is expected to provide one of the first empirical descriptions of a structured transition pathway from our region and to inform the future development of standardized, disease-appropriate transition frameworks for pediatric-onset metabolic bone disorders.

## 2. Materials and Methods

### 2.1. Study Design and Setting

This single-center, retrospective cohort study was conducted at a tertiary pediatric–adult metabolic bone clinic, utilizing a structured transition framework. The transition infrastructure consists of multidisciplinary team meetings (MDTM), a joint pediatric–adult transition clinic, and subsequent follow-up within adult endocrinology services. Medical records, MDTM notes, and adult clinic documentation were systematically reviewed to characterize engagement and outcomes during the transition period.

### 2.2. Study Population and Eligibility Criteria

Patients were eligible if they had a confirmed diagnosis of a pediatric-onset metabolic bone disease—including osteogenesis imperfecta, hypophosphatemic rickets, hypoparathyroidism, hypophosphatasia, McCune–Albright syndrome, or vitamin D-dependent rickets—were aged ≥17 years at the time of transition evaluation, had received at least two years of pediatric follow-up, and had been reviewed by the MDTM for transfer planning.

Patients were excluded if their medical records were incomplete or if they were not formally considered for transition despite reaching an appropriate age. Conditions outside the spectrum of metabolic bone disease were also excluded.

### 2.3. Transition Pathway and Clinical Workflow

The structured transition model comprised three interconnected steps.

*Multidisciplinary Team Meeting (MDTM)*

The transition process was primarily facilitated through multidisciplinary team meetings (MDTM) followed by a joint pediatric–adult transition clinic. Patients were considered for transition after reaching the appropriate age, achieving clinical stability, completing the diagnostic evaluation, and maintaining regular pediatric follow-up. Beginning at approximately 17 years of age, patients and their families were informed about the transition process during routine pediatric endocrinology visits, and the decision to initiate transition was made jointly by the pediatric endocrinology team, the patient, and the family.

MDTMs were held on a regular basis and attended by pediatric and adult endocrinologists together with other relevant specialists, including orthopedic surgeons, metabolic disease specialists, dietitians, and physiotherapists when required. Before each meeting, the pediatric endocrinology team prepared a detailed clinical summary for each patient, including diagnosis, previous treatments, current disease status, comorbidities, and transition readiness. During the meeting, the multidisciplinary team reviewed the patient’s clinical course, discussed anticipated disease-specific challenges in adult care, evaluated ongoing treatment requirements, and formulated individualized recommendations regarding follow-up and long-term management. A written individualized summary was prepared for each patient. The summary also included recommendations regarding treatment continuation or modification, follow-up scheduling, and additional specialty consultations when required to facilitate continuity of care after transfer.

*Joint Pediatric–Adult Transition Clinic*

Following the MDTM, patients attended a joint pediatric–adult transition clinic in which both pediatric and adult endocrinologists participated. Patients were encouraged to attend together with their families whenever possible. During this visit, medical information was formally transferred, long-term disease management was discussed, and patients were educated regarding treatment adherence, self-management, and the importance of continued follow-up after transfer to adult care. Disease-specific issues, including reproductive health or mobility issues when appropriate, were also addressed. This joint consultation constituted the formal initiation of the transfer to adult care.

Patients who were unable to attend the joint clinic were subsequently referred to adult endocrinology, as their clinical information and management plan had already been reviewed during the MDTM.

*Adult Endocrinology Follow-Up*

After transfer, patients were followed by the adult endocrinology team. Follow-up visits were scheduled according to the patient’s diagnosis and clinical needs during the first year after transfer. Documentation of visit frequency, treatment modifications, laboratory monitoring, and continuity of care was collected during the first 12 months. Follow-up adherence was assessed through documented clinic attendance and continuity of care records in the electronic medical record.

### 2.4. Data Collection and Variables

Demographic, clinical, and transition-related variables were systematically extracted from electronic medical records and MDTM documentation. Variables were categorized as follows:

*Demographic variables*

• Age at transition evaluation.
• Sex.

*Disease-specific variables*

• Primary diagnosis and diagnostic subtype.
• Age at initial diagnosis.

*Treatment variables*

• Previous disease-specific therapies.
• Treatment modifications during the first year after transfer.

*Transition-related variables*

• Attendance at multidisciplinary team meeting (MDTM).
• Participation in the joint pediatric–adult transition clinic.
• Completion of transfer to adult endocrinology.
• Number of adult endocrinology clinic visits within 12 months.
• Lost-to-follow-up status.

Two reviewers independently performed data abstraction and verification to ensure the accuracy and completeness of the data.

### 2.5. Outcomes

The primary outcome was successful transition, defined as attending at least one adult endocrinology visit within 12 months of transfer.

Secondary outcomes included attendance at the joint transition clinic, regular follow-up in the adult clinic during the preceding 12 months, treatment continuity, therapy modification rates, and diagnostic subgroup differences in transition engagement.

### 2.6. Statistical Analysis

Statistical analyses were primarily descriptive because of the small sample size and the heterogeneity of diagnostic subgroups. Continuous variables were summarized as median (range) or mean ± standard deviation, as appropriate. Categorical variables were presented as frequencies and percentages. Analyses were performed using IBM SPSS Statistics for Windows, version 29.0 (IBM Corp., Armonk, NY, USA).

### 2.7. Ethical Considerations

The study protocol was approved by the institutional ethics committee of Bursa Uludağ University Faculty of Medicine (approval number: 2023-23/24). As a retrospective study using anonymized data, the requirement for informed consent was waived in accordance with institutional guidelines.

## 3. Results

### 3.1. Patient Characteristics

Twenty adolescents and young adults with pediatric-onset metabolic bone disorders were included in the transition program. The median age at transition assessment was 18.4 years (range, 17.0–19.3). Diagnoses included osteogenesis imperfecta (*n* = 4), McCune–Albright syndrome (*n* = 3), postoperative hypoparathyroidism (*n* = 3), hypophosphatemic rickets (*n* = 2), vitamin D-dependent rickets type 1 (*n* = 2), DiGeorge syndrome (*n* = 2), idiopathic hypoparathyroidism (*n* = 2), pseudohypoparathyroidism (*n* = 1), and familial hypocalciuric hypercalcemia (*n* = 1). Transition ages were comparable across diagnostic categories, with medians ranging between 17.8 and 19.2 years (Table 1).

### 3.2. Engagement in the Transition Pathway

**Figure 1:**
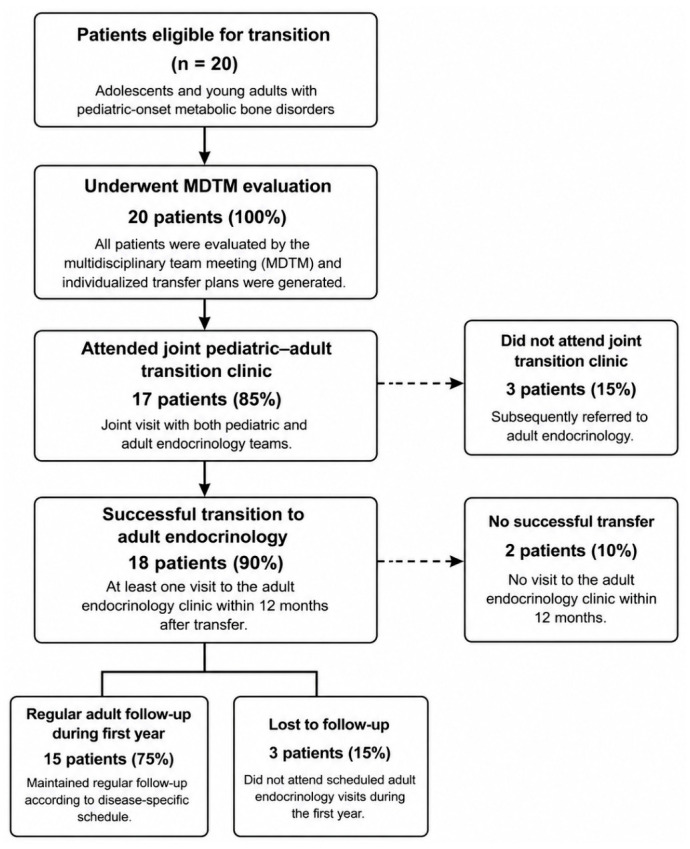
Patient flow through the structured multidisciplinary transition pathway for adolescents and young adults with pediatric-onset metabolic bone diseases. All patients underwent MDTM evaluation, followed by assessment of attendance at the joint pediatric–adult transition clinic, successful transfer to adult endocrinology, and follow-up during the first year after transition.

All patients underwent multidisciplinary team meeting (MDTM) evaluation, during which individualized transfer plans were generated. The overall patient flow through the structured transition pathway is summarized in Figure 1. Attendance at the joint pediatric–adult transition clinic was high, with 85% (17/20) of participants present. Participation was complete among patients with OI, MAS, VDDR type 1, and DiGeorge syndrome, while attendance was lower in postoperative hypoparathyroidism and in the single case of pseudohypoparathyroidism.

One patient who did not attend the joint transition clinic subsequently attended the adult endocrinology clinic and therefore met the predefined criterion for successful transition. Abbreviation: MDTM, multidisciplinary team meeting.

### 3.3. Transition Completion and Adult Follow-Up

Successful completion of transition—defined as at least one visit to the adult endocrinology clinic within 12 months—was observed in 18 of 20 patients (90%). Sustained engagement, as reflected by regular follow-up in the past year, occurred in 75% (15/20) of cases. Follow-up retention varied across diagnostic groups. Patients with OI, MAS, XLH, VDDR type 1, and DiGeorge syndrome demonstrated uninterrupted adult follow-up. In contrast, postoperative hypoparathyroidism and idiopathic hypoparathyroidism showed greater variability in visit adherence, and the patient with familial hypocalciuric hypercalcemia did not return for follow-up.

### 3.4. Treatment Modification After Transfer

Treatment modifications were individualized according to disease status, biochemical assessment, current therapy, and multidisciplinary clinical judgment rather than predefined protocol-driven criteria. Reassessment in the adult clinic resulted in treatment adjustments for 40% of patients who transitioned during the first year. Modifications included optimization of phosphate and calcitriol therapy, an updated evaluation of bisphosphonate exposure, and reassessment of long-term therapeutic needs, such as continuing burosumab in XLH. Notably, no acute complications, hospitalizations, or disease-related emergencies occurred during the transition period.

### 3.5. Lost to Follow-Up

Three patients (15%) were lost to follow-up after transfer. These individuals generally had milder clinical or biochemical profiles. Although no single diagnosis predicted loss-to-follow-up, postoperative hypoparathyroidism and familial hypocalciuric hypercalcemia were represented within this subgroup.

## 4. Discussion

This study provides one of the first real-world evaluations of a structured transition pathway for adolescents and young adults with pediatric-onset metabolic bone disorders. Our findings suggest that implementation of a structured multidisciplinary transition pathway was feasible and was accompanied by high transition completion rates (90%) and favorable short-term retention in adult care (75%), particularly among individuals with complex or syndromic conditions, such as osteogenesis imperfecta (OI), McCune–Albright syndrome (MAS), and hypophosphatemic rickets (XLH). These results contrast with the markedly lower transition success reported in chronic pediatric conditions such as epilepsy, inflammatory bowel disease, and pediatric-onset lupus, where loss to follow-up rates between 20% and 50% are common [12,13].

The strong engagement observed in our cohort may reflect the unique clinical characteristics of metabolic bone diseases, which are lifelong disorders with persistent skeletal, biochemical, and functional complications extending well into adulthood. As highlighted in recent reviews, individuals with OI experience a second peak in fracture risk in adulthood and may develop mobility limitations, chronic pain, or progressive deformities despite early bisphosphonate therapy [1,3]. Patients with XLH frequently continue to exhibit enthesopathy, osteomalacia-related symptoms, and functional impairment, while adults with hypophosphatasia risk misdiagnosis and iatrogenic harm from inappropriate antiresorptive therapies [6,7].

These disease characteristics may partly explain the favorable engagement observed in our cohort; however, this interpretation remains speculative and requires confirmation in larger comparative studies.

Another observation in this small cohort is the diagnostic variability in follow-up patterns. Although patients with OI, MAS, XLH, vitamin D-dependent rickets, and DiGeorge syndrome demonstrated complete retention in adult care, these findings should be interpreted cautiously because of the very small numbers within each diagnostic subgroup. These conditions often involve multisystem complexity or require ongoing pharmacological management, which may contribute to adherence [5,7]. By contrast, postoperative hypoparathyroidism and idiopathic hypoparathyroidism showed greater variability in visit continuity. Compared with skeletal dysplasias or phosphate-wasting disorders, hypoparathyroidism may sometimes be perceived as more “stable” or “medically manageable,” potentially reducing the perceived need for follow-up despite the risk of chronic complications such as hypocalcemia, neurocognitive symptoms, or renal calcifications [7]. Our observations are consistent with reports from epilepsy and IBD cohorts, particularly in adolescents who report emotional dependence on pediatric providers or low confidence in managing their own condition [13].

Our findings further support the potential value of a structured multidisciplinary transition model. Evidence from systematic reviews and specialty-specific reviews suggests that coordinated transition interventions improve adherence, clinic attendance, and disease-related outcomes across chronic conditions [8,11,14].

The MDTM in our study ensured individualized transition planning, cross-specialty communication, and harmonized expectations between pediatric and adult teams—elements that have been repeatedly identified as essential for preventing gaps in care [8,9]. In pediatric-onset lupus, for example, inadequate coordination between pediatric and adult rheumatologists has been associated with prolonged lapses in follow-up and increased disease activity after transfer [12]. Structured approaches, including shared-care visits and transition coordinators, significantly reduce these adverse outcomes [5,11].

Similar principles may have contributed to the favorable transition outcomes observed in our cohort. Treatment continuity also emerged as a relevant theme. Nearly 40% of our patients required therapeutic modification during the first year of adult follow-up. This aligns with the literature, which indicates that metabolic bone conditions evolve through adolescence and adulthood, necessitating a dynamic reassessment of phosphate metabolism, bone turnover, mobility, reproductive endocrinology, and cardiovascular health [5,7,15]. In XLH, restricted access to burosumab in adulthood—also reflected in our cohort—represents a well-recognized barrier that underscores the need for harmonized pediatric–adult treatment policies [16,17]. Treatment gaps during transition have been similarly documented in IBD and epilepsy, where changes in insurance coverage, medication availability, or prescriber practices can disrupt continuity of care [13].

Despite a high overall success rate, 15% of patients were lost to follow-up, primarily those with milder biochemical abnormalities or a lower perceived disease burden. This is consistent with broader transition literature, which shows that patients with fewer symptoms, stable biomarkers, or a limited understanding of future risks are more likely to disengage from adult services [11,14]. In chronic diseases such as epilepsy and IBD, emotional dependence on pediatric providers, fear of the adult system, and insufficient self-management skills have been identified as strong predictors of drop-out [12,13]. These findings highlight the need for targeted educational interventions that emphasize long-term risks, even in conditions perceived as “stable.”

The present study fills a notable gap in the literature. Whereas transition models have been extensively studied in rheumatology, gastroenterology, and neurology, data on metabolic bone disorders remain sparse [7,18]. Most available publications focus on pediatric management, natural history, or adult manifestations, with little empirical evaluation of the transfer process itself. Our study provides foundational data supporting the feasibility of a structured transition model for rare metabolic bone diseases. Nevertheless, the modest sample size and single-center design limit generalizability, and longer-term outcomes—including fracture rates, biochemical stability, functional mobility, and adherence beyond the first year—remain to be explored [1,3,4].

This study has several limitations. First, the sample size was modest, reflecting the rarity of pediatric-onset metabolic bone disorders and the single-center design. Although the cohort allowed for descriptive comparisons across diagnostic subgroups, it limited the ability to perform more robust statistical analyses or to explore predictors of transition success. Second, the study included only patients who had entered the structured MDTM-based transition pathway and were formally evaluated for transfer to adult care. Patients who never reached the transition process, declined participation, or discontinued pediatric follow-up before referral were not captured in the present cohort. Consequently, selection bias cannot be excluded, and the observed transition completion and follow-up rates may overestimate those achievable in an unselected population of adolescents with pediatric-onset metabolic bone diseases. In addition, the absence of a comparator group precludes any conclusions regarding the effectiveness of the transition program compared with standard care. Therefore, the present findings should be interpreted as demonstrating the feasibility of the structured transition pathway rather than its comparative effectiveness. Third, follow-up was restricted to the first year after transfer; longer-term outcomes, such as fracture incidence, biochemical stability, mobility, and treatment adherence, were not evaluated and should be examined in future studies. Fourth, because the data were derived from a retrospective chart review, they are inherently dependent on documentation quality, which may underestimate certain clinical or psychosocial factors influencing transition readiness. Finally, disease-specific subgroup analyses should be interpreted with caution because each diagnostic subgroup included only a small number of patients. Accordingly, subgroup comparisons were intended to provide descriptive, hypothesis-generating observations rather than definitive conclusions regarding disease-specific transition outcomes.

Despite these limitations, this exploratory study provides preliminary real-world data on transition outcomes in adolescents and young adults with rare metabolic bone diseases and may help inform future multicenter prospective studies and the development of disease-specific transition strategies. Future multicenter studies should also focus on developing and validating disease-specific transition algorithms, including standardized recommendations for clinical assessment, treatment planning, and long-term follow-up for individual metabolic bone disorders.

## 5. Conclusions

In conclusion, our findings suggest that a structured multidisciplinary transition pathway is feasible and may facilitate successful transfer and short-term follow-up among adolescents and young adults with rare metabolic bone diseases. Diagnostic subgroups show differing patterns of engagement, suggesting that transition readiness and disease perception should be tailored to condition-specific needs. These findings provide a foundation for future multicenter comparative studies evaluating structured transition frameworks for rare metabolic bone disorders.

## Figures and Tables

**Table 1:** Characteristics of Patients with Metabolic Bone Diseases Evaluated During Transition.

Diagnosis	*n*	Median Age at Transition (Years)	Attended Initial Transition Clinic	Regular Follow-Up	Seen Within Last Year
**Osteogenesis imperfecta**	4	18.5 (18.08–19.33)	3	4	4
**McCune–Albright** **syndrome**	3	18.5 (18.0–19.00)	3	2	3
**Postoperative hypoparathyroidism**	3	18.08 (18.00–18.25)	1	2	2
**Hypophosphatemic** **rickets**	2	17.83 (17.08–18.58)	1	2	2
**Vitamin D-dependent** **rickets type 1**	2	17.83 (17.66–18.00)	2	2	2
**DiGeorge syndrome**	2	18.62 (18.16–19.08)	2	2	2
**Hypoparathyroidism**	2	18.1 (18.00–18.20)	1	1	2
**Pseudohypoparathyroidism**	1	18.40	0	1	1
**Familial hypocalciuric** **hypercalcemia**	1	19.16	0	0	0

**Note:** Data are presented as the number of patients (*n*), unless otherwise indicated.

## Data Availability

The data supporting the findings of this study are not publicly available due to privacy and ethical restrictions but are available from the corresponding author upon reasonable request.

## Abbreviations

BMD	Bone mineral density
DXA	Dual-energy X-ray absorptiometry
FHH	Familial hypocalciuric hypercalcemia
HPP	Hypophosphatasia
MAS	McCune–Albright syndrome
MDTM	Multidisciplinary team meeting
OI	Osteogenesis imperfecta
PTH	Parathyroid hormone
T1DM	Type 1 diabetes mellitus
VDDR	Vitamin D-dependent rickets
XLH	X-linked hypophosphatemic rickets
